# A recombinant CYP11B1 dependent *Escherichia coli* biocatalyst for selective cortisol production and optimization towards a preparative scale

**DOI:** 10.1186/s12934-015-0209-5

**Published:** 2015-02-25

**Authors:** Lina Schiffer, Simone Anderko, Anna Hobler, Frank Hannemann, Norio Kagawa, Rita Bernhardt

**Affiliations:** Department of Biochemistry, Saarland University, 66123 Saarbrücken, Germany

**Keywords:** Cortisol, Human CYP11B1, Steroid biotransformation, Whole-cell biocatalysis, *E. coli*

## Abstract

**Background:**

Human mitochondrial CYP11B1 catalyzes a one-step regio- and stereoselective 11β-hydroxylation of 11-deoxycortisol yielding cortisol which constitutes not only the major human stress hormone but also represents a commercially relevant therapeutic drug due to its anti-inflammatory and immunosuppressive properties. Moreover, it is an important intermediate in the industrial production of synthetic pharmaceutical glucocorticoids. CYP11B1 thus offers a great potential for biotechnological application in large-scale synthesis of cortisol. Because of its nature as external monooxygenase, CYP11B1-dependent steroid hydroxylation requires reducing equivalents which are provided from NADPH via a redox chain, consisting of adrenodoxin reductase (AdR) and adrenodoxin (Adx).

**Results:**

We established an *Escherichia coli* based whole-cell system for selective cortisol production from 11-deoxycortisol by recombinant co-expression of the demanded 3 proteins. For the subsequent optimization of the whole-cell activity 3 different approaches were pursued: Firstly, *CYP11B1* expression was enhanced 3.3-fold to 257 nmol∗L^−1^ by site-directed mutagenesis of position 23 from glycine to arginine, which was accompanied by a 2.6-fold increase in cortisol yield. Secondly, the electron transfer chain was engineered in a quantitative manner by introducing additional copies of the *Adx* cDNA in order to enhance *Adx* expression on transcriptional level. In the presence of 2 and 3 copies the initial linear conversion rate was greatly accelerated and the final product concentration was improved 1.4-fold. Thirdly, we developed a screening system for directed evolution of CYP11B1 towards higher hydroxylation activity. A culture down-scale to microtiter plates was performed and a robot-assisted, fluorescence-based conversion assay was applied for the selection of more efficient mutants from a random library.

**Conclusions:**

Under optimized conditions a maximum productivity of 0.84 g cortisol∗L^−1^∗d^−1^ was achieved, which clearly shows the potential of the developed system for application in the pharmaceutical industry.

**Electronic supplementary material:**

The online version of this article (doi:10.1186/s12934-015-0209-5) contains supplementary material, which is available to authorized users.

## Background

Cortisol, the major human glucocorticoid, plays a crucial role in the physiological adaption to stress, the regulation of energy mobilization and immune response [1]. Its anti-inflammatory and immunosuppressive effects render it a powerful agent for the abatement of classical inflammatory symptoms like pain or swelling that occur in the course of acute and chronic inflammatory or autoimmune diseases. Moreover, cortisol serves as an intermediate in the production of synthetic glucocorticoids, which can exhibit even greater glucocorticoid effects but less mineralocorticoid side effects. Prednisolone, for example, is derived from cortisol by a microbial 1,2-dehydrogenation [2]. The hydroxyl group in position 11β of the cortisol molecule and its synthetic derivatives is the key functionalization that provides its glucocorticoid effects. It is the same functionalization that is the most difficult one to be introduced chemically or microbially in the preparative synthesis of cortisol. In current industrial production it is carried out as the final step of a hemi synthesis by microbial transformation of 11-deoxycortisol with fungal cultures of the genus *Curvularia* in a scale of about 100 tons per year [3] by taking advantage of the organism’s endogenous steroid 11β-hydroxylase activity [4]. However, this process suffers from poor selectivity. Purification and characterization of the responsible enzyme revealed low regioselectivity as 11β-hydroxylation of the substrate is accompanied by 14α-hydroxylation [5,6]. Consecutively, it is of great interest to develop alternative biocatalysts for a more selective and efficient introduction of the 11β-hydroxyl group into synthetic glucocorticoids.

**Figure 1:**
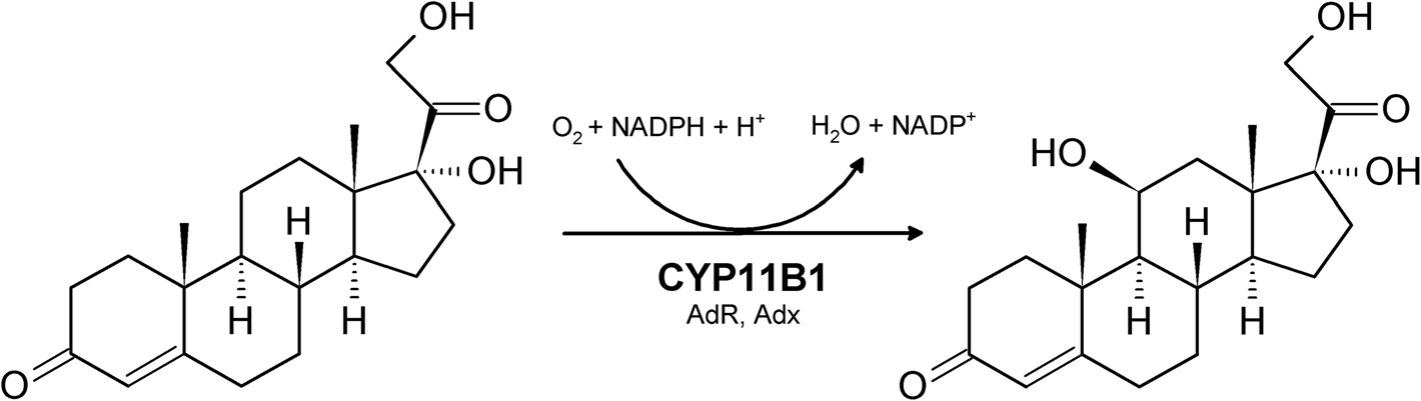
**Scheme of the CYP11B1-catalyzed 11** **β-hydroxylation of 11-deoxycortisol yielding cortisol.** Electrons are transferred from NADPH via AdR and Adx to CYP11B1, which activates molecular oxygen and incorporates one oxygen atom into the steroid substrate by means of a hydroxylation in a regio- and stereoselective manner, while the other oxygen is reduced to water.

In the human adrenal cortex, which represents the principal tissue for the biosynthesis and secretion of gluco- and mineralocorticoids, cortisol is formed selectively from 11-deoxycortisol by the 11β-hydroxylase CYP11B1 (human steroid 11β-hydroxylase) (Figure 1) [7-10]. Hence, in the context of cortisol production CYP11B1 also constitutes an attractive candidate for a biotechnological application.

CYP11B1 belongs to the evolutionary highly conserved superfamily of cytochromes P450 (P450). P450s catalyze versatile biotransformations of a wide range of substrates in all domains of life. It is mainly their capability to activate molecular oxygen and to incorporate one oxygen atom into a substrate molecule leading to a regio- and stereoselective hydroxylation that vests them a tremendous biotechnological potential in the synthesis of pharmaceuticals and fine chemicals [11-14]. Due to their nature as external monooxygenases, P450s require an external electron donor, which is in general NAD(P)H, and an electron delivering system composed of one or more additional proteins [15]. In case of CYP11B1, which represents a mitochondrial P450, the respective electron transfer chain is constituted of AdR, an NADPH-dependent flavoprotein, and Adx, a [2Fe-2S]-cluster protein that interacts with the P450. Such complexity of P450 systems along with the necessity of a costly cofactor is so far one of the determining factors that restrict the employment of P450 catalysts in a larger scale. The most promising approach to overcome these limitations is the employment of whole-cell systems that offer cofactors from their metabolism and a cellular environment for the support of protein stability and do not require time-consuming purification steps [12,16]. The current state of molecular biology and recombinant protein expression enables the exploitation of different microbial hosts for application of biotechnologically interesting enzymes. CYP11B1 could already be applied in engineered yeast strains (*Saccharomyces cerevisiae* and *Schizosaccharomyces pombe*) that convert 11-deoxycortisol to cortisol [17-19] or even accept simple carbon sources as substrate when additional sterol providing and modifying genes are engineered and introduced [20]. However, optimization of these systems towards a relevant scale is a great challenge. Our laboratory previously reported the first expression of *CYP11B1* in a bacterial host (*Escherichia coli*, *E. coli*) for purification and enzymatic characterization [21]. Subsequently, we decided to use this fast-growing and genetically amenable microorganism, which does not possess any endogenous, by-product generating P450s, and established the first bacterial whole-cell system for application of CYP11B1 in cortisol preparative scale biosynthesis. The entire redox chain consisting of AdR, Adx and the P450 was introduced into *E.coli*. For optimization, CYP11B1 expression was enhanced by site-directed mutagenesis, the co-expression of Adx was quantitatively adjusted on transcriptional level and CYP11B1 was engineered by molecular evolution towards higher activity.

## Results

### Establishment of a CYP11B1 based whole-cell system for cortisol synthesis in *E. coli*

In order to employ human CYP11B1 for steroid hydroxylation in *E. coli*, we created the plasmid Twin_11B1 which is based on the pET-17b vector and carries the cDNAs of human CYP11B1 including the modifications described in the Material and methods section, bovine AdR and bovine Adx in a tricistronic transcription unit separated by ribosomal binding sites. This enables the reconstitution of a functional P450 system in the host organism. The *E. coli* strain C43(DE3), which has previously been reported as advantageous for the synthesis of membrane proteins [22], was co-transformed with the new plasmid and the chaperone encoding plasmid pGro12 [23]. Chaperone synthesis supports the proper folding of membrane proteins in the prokaryotic host [21,24-26]. Protein production was carried out in a complex medium and could be confirmed by Western Blot analysis with primary antibodies raised against CYP11B1, AdR and Adx, respectively.

**Figure 2:**
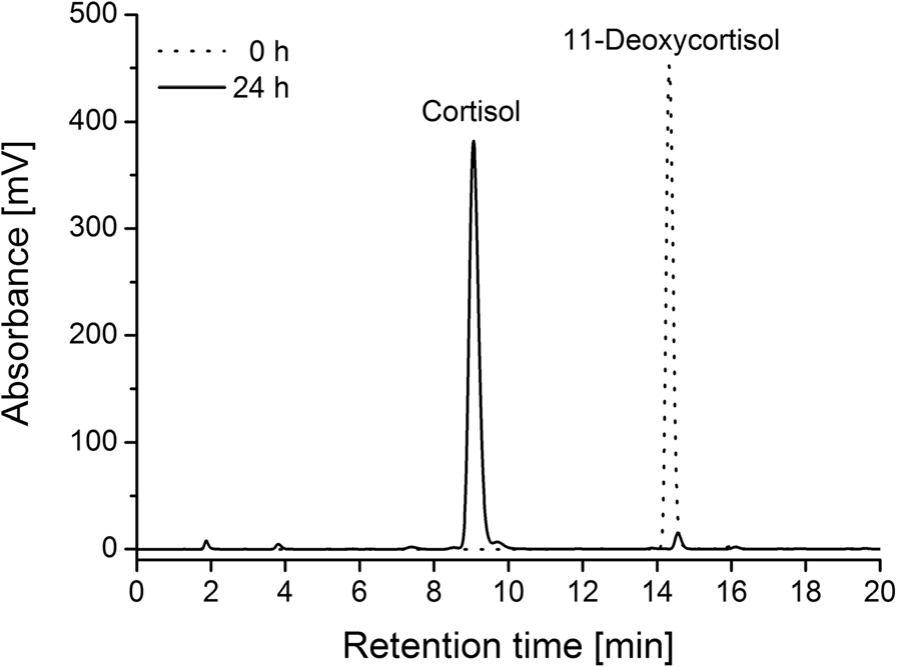
**HPLC chromatogram of the CYP11B1 dependent conversion of 11-deoxycortisol to cortisol by recombinant** ***E. coli*** **.** Chromatograms were obtained from extracted samples of *E. coli* cultures after 0 h (dotted line) and 24 h (solid line) of incubation after transfer into potassium phosphate buffer and addition of 1.2 mM substrate. Substance peaks obtained with an acetonitrile/water gradient represent cortisol with a retention time of 9.0 min and 11-deoxycortisol with a retention time of 14.3 min.

Subsequent transformation of 11-deoxycortisol was conducted with non-growing cells in buffer supplemented with glycerol as carbon source to ensure a sufficient availability and regeneration of NADPH for the P450 reaction [27]. Thereby, a fixed cell density of 25 g_wcw_/L was adjusted in all experiments. HPLC analysis of extracts from the resting cells demonstrates a selective CYP11B1 dependent 11β-hydroxylation of 11-deoxycortisol yielding cortisol (Figure 2). Steroids were identified via their retention times in comparison with standards from commercial sources.

As the solubilty of steroidal compounds can be a limiting factor for their bioconversion [12], we subsequently evaluated the effect of different dissolving agents for the addition of 11-deoxycortisol on the activity of the new whole-cell system. Each agent was added to a final concentration of 6% (vol/vol). While the employment of ethanol lead to the lowest cortisol yield and cyclodextrines and a 1:1 mixture of EtOH and PEG-400 slightly improved the final yield, the best results were obtained with DMSO, which was consecutively used for substrate supply in all subsequent experiments.

### Optimization of CYP11B1 expression

**Figure 3:**
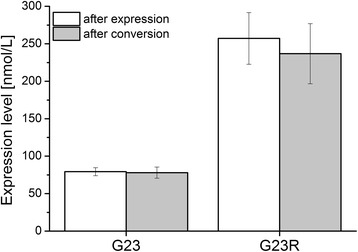
**Expression level of CYP11B1 enzyme variants carrying either glycine (G23) or arginine (G23R) in position 23.** Recombinant *E. coli* cultures expressing either wild type or mutant were harvested after the expression period (white bars) or at the end of substrate conversion (grey bars) under the conditions described in Material and methods. P450 concentration was determined by CO-difference spectroscopy with the supernatant of the cell lysate after ultracentrifugation. Values represent the mean of three experiments with the respective standard deviation.

In order to improve the *CYP11B1* expression in *E. coli* and thus the activity of the recombinant system, we performed site-directed mutagenesis in position 23 of the *E. coli* adapted sequence. Glycine, which corresponds to the published wildtype amino acid in that position [28,29], was replaced by the hydrophilic amino acid arginine. An analogous replacement from glycine to arginine, which was introduced into human *CYP19* when performing N-terminal replacements with related sequences from other P450s by Kagawa et al. [30], was reported to significantly enhance the expression in *E. coli.* The corresponding residue in human CYP11B1 was identified by primary sequence alignment. The expression level of the 2 CYP11B1 variants was estimated by CO-difference spectroscopy (Figure 3). The introduction of the arginine residue could succesfully enhance CYP11B1 level from 79 to 257 nmol∗L^−1^ and no significant reduction over the conversion period could be observed.

**Figure 4:**
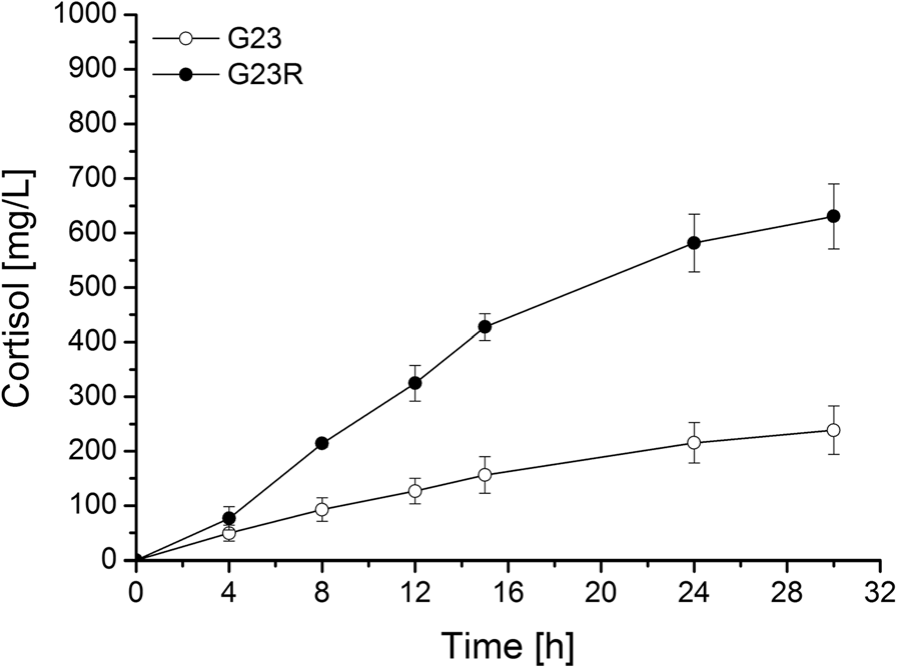
**Comparison of the whole-cell cortisol formation by CYP11B1 enzyme variants carrying either glycine (G23) or arginine (G23R) in position 23 in recombinant** ***E. coli*** **.** Reactions were performed after a 21-h expression period in TB medium with resting cells in the presence of 6% DMSO and 3 mM 11-deoxycortisol. Extracted steroids were analyzed by RP-HPLC. Values represent the mean of three conversion experiments conducted in parallel with respective standard deviation.

Cortisol formation by both enzyme variants in the whole-cell system was monitored in a time-dependent manner. In general, the system exhibited a linear volumetric productivity in an initial phase of at least 12 h. Afterwards the velocity of cortisol formation decreased and a final product concentration was reached after 30 h. The deployment of CYP11B1 G23R for the whole-cell conversion of 11-deoxycortisol increased the final cortisol yield after 30 h by a factor of 2.6 compared with the G23 variant from 239 to 631 mg∗L^−1^ (Figure 4). The initial linear productivity was enhanced in the same range from 11 to 27 mg∗L^−1^∗h^−1^. Therefore, CYP11B1 G23R is applied for all further experiments and will be termed Pa1 (Parent generation 1) in the following molecular evolution studies.

### Influence of Adx copy number

**Figure 5:**
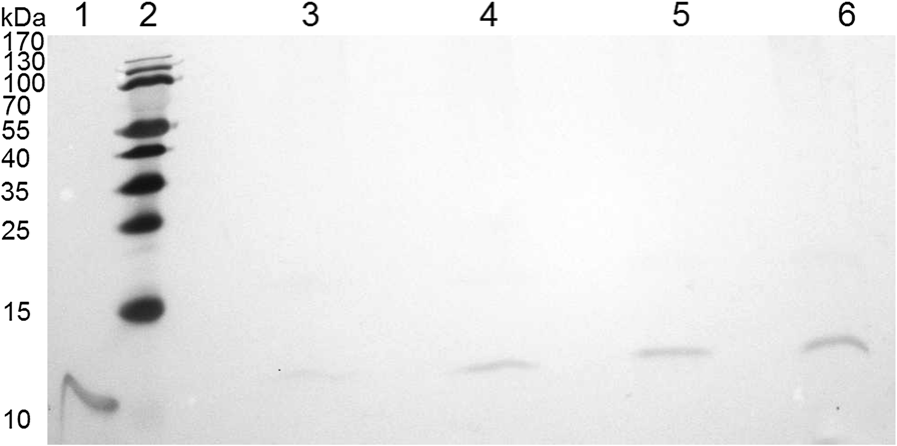
**Western blot analysis of Adx expression by recombinant** ***E. coli*** **cells.** *Lane 1* shows purified bovine Adx_4–108_ (~11,8 kDa). *lane 2* shows a pre-stained protein marker with molecular weights indicated on the left of the picture. *Lanes 3* to *6* represent the lysate of *E. coli* cells expressing the CYP11B1 redox system from variants of the Twin_11B1 plasmid carrying 1 to 4 copies of bovine Adx_1–108_ (~12,0 kDa). Adx detection was carried out with polyclonal antibodies and a peroxidase-based color reaction. The relative expression level of Adx was determined by measuring the band intensity with the Image Lab software.

As electron supply frequently constitutes the limiting step in the efficiency of P450 systems [31-33], our next approach for improving the whole-cell activity of CYP11B1 was an increase of the amount of Adx in the system, in order to enhance electron transfer to CYP11B1. For that, we constructed variants of the expression plasmid Twin_11B1 with up to 4 copies of the Adx cDNA by successively integrating additional copies including a 5′-ribosomal binding site at the end of the trancription unit according to Blachinsky et al. [34]. The relative increase of *Adx* expression was estimated by Western Blot (Figure 5) and evaluation of the Adx signal with an imaging software. With the introduction of a second cDNA copy *Adx* expression was increased approximately 2.4-fold and a maximum of *Adx* expression (3.3-fold increase) was reached with the insertion of a third copy which could not be further augmented by a fourth copy. No influence on *CYP11B1* expression was observed by CO-difference spectroscopy, when 2 or 3 copies of *Adx* were present on the expression vector. With the insertion of a fourth *Adx* copy, the CYP11B1 titer was, however, reduced by 50% to approximately 120 nmol∗L^-1^ and the construct was thus not further investigated.

**Figure 6:**
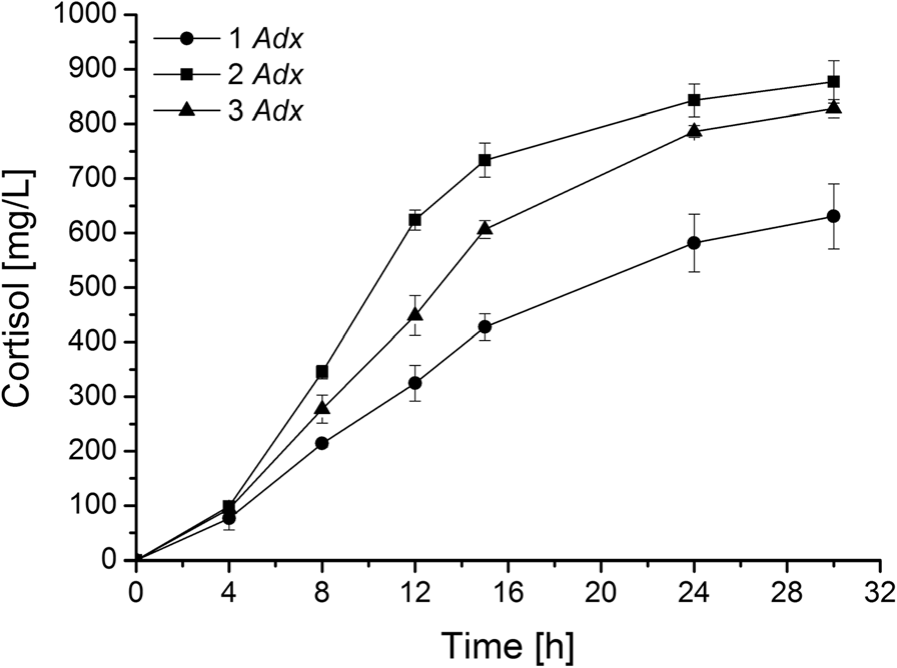
**Comparison of the cortisol formation by recombinant** ***E. coli*** **transformed with different versions of the P450 system encoding plasmid Twin_11B1 with 1 (circles), 2 (squares) or 3 (triangles) copies of the Adx cDNA.** Reactions were performed after a 21-h expression period in TB medium with resting cells in the presence of 6% DMSO and 3 mM 11-deoxycortisol. Extracted steroids were analyzed by RP-HPLC. Values represent the mean of three conversion experiments conducted in parallel with respective standard deviation.

Subsequent whole-cell conversions conducted with 2 and 3 *Adx* copies increased the final product concentration from 631 mg∗L^−1^ for the initial system with 1 Adx to 877 and 828 mg∗L^−1^, respectively (Figure 6). Although both plasmids enabled a comparable final yield, the presence of 2 Adx copies also greatly enhanced the initial productivity over the first 12 h from 27 to 52 mg∗L^−1^∗h^−1^, while a third copy diminished this rate again to 37 mg∗L^−1^∗h^−1^ but exhibited a longer phase of linearity in time-dependent product formation leading to a comparable final yield.

### Development of a screening system for CYP11B1 activity

**Figure 7:**
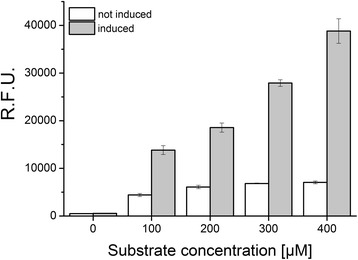
**Relative fluorescence developed by recombinant** ***E. coli*** **cultures after incubation with different 11-deoxycortisol concentrations.** Cells were transformed with Twin_11B1 and pGro12 and incubated with 11-deoxycortisol for 48 h in TB medium containing 1 mM δ-Ala (white bars) that has been additionally supplemented with 1 mM IPTG and 4 mg/mL arabinose for induction (grey bars). Steroid specific fluorescence was generated by the described assay and measured in relative units (R.F.U.) at λ_ex_ 485 nm and λ_em_ 535 nm. Values represent the mean of triplicates with standard deviations.

For further improvement of the CYP11B1 activity using molecular evolution, we adapted the biotransformation with the *E. coli* whole-cell system and its subsequent evaluation to a microtiter plate format which enables a high and robot-assisted throughput. Culture size was scaled down to 1 mL and substrate conversion was carried out in TB medium with inoculation, induction of protein expression and addition of the substrate 11-deoxycortisol at the same time in order to reduce working steps to a minimum. For evaluation of enzyme activity, we employed a fluorescence assay, which makes use of the fluorescence developed by steroids with an intensity in dependence on the substitution of the steran scaffold [35-37]. As cortisol exposes a higher fluorescence than 11-deoxycortisol due to the additional hydroxyl group that is introduced in position 11β, enzyme variants with an increased hydroxylation activity can easily be selected. In order to ensure optimal conditions for the detection of mutants with improved activity, conversion with the parental enzyme variant Pa1 (CYP11B1 G23R) was tested with different substrate concentrations and the fluorescence was determined after 48 h of incubation in comparison with control cultures that were incubated with the respective 11-deoxycortisol concentration but without induction of protein expression. A clear difference between unspecific and steroid specific fluorescence could be observed as well as a significant increase of the relative fluorescence by a factor of more than 3 upon induction of protein expression, which proofs the presence of an active P450 system and thus the applicability of the system for monitoring 11-deoxycortisol conversion to cortisol (Figure 7).

Higher substrate concentrations result in an increased activity, which is displayed by an increased fluorescence signal and which was confirmed by HPLC analysis. Steroids were identified by their retention time and relative quantification depicted 20.2 ± 2.9% conversion of 11-deoxycortisol to cortisol at a concentration of 400 μM, which was subsequently chosen for the selection of activity improved mutants.

### Generation and screening of a CYP11B1 mutant library

**Figure 8:**
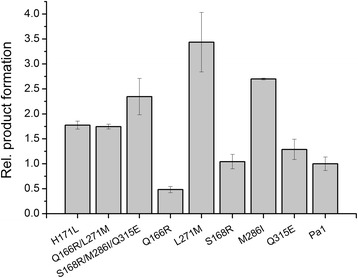
**Relative cortisol formation of CYP11B1 enzyme variants in microtiter plates evaluated by HPLC.** Mutants H171L, Q166R/L271M and S168R/M286I/Q315E were selected in the fluorescence based screening of a random mutant library. All other mutants were created by site-directed mutagenesis. Conversion took place for 48 h as described under Material and methods with 400 μM 11-deoxycortisol and cortisol formation was evaluated by HPLC and is presented in a relative manner as mean of triplicates with respective standard deviation. Product formation of CYP11B1 Pa1 was assigned as 1.

A random CYP11B1 mutant library was created by epPCR applying the sequence of CYP11B1 Pa1 as a template. The emerged sequence variants were cloned into the Twin plasmid and C43(DE3) *E. coli* cells were co-transformed with the mutant Twin_11B1 plasmid library as well as pGro12 and were spread on agar plates. Sequencing of 10 randomly picked clones revealed a mutational frequency of 2.83 base exchanges per kilobase. Approximately 1000 clones were screened with the fluorescence assay for enhanced hydroxylation activity towards 11-deoxycortisol. From these, 53 clones, which exhibited an at least 1.5-fold higher fluorescence signal than the CYP11B1 Pa1 control incubated on the same plate, were re-screened in triplicates. For 3 mutants, which still showed an average increase of the fluorescence signal by more than the 1.5-fold, an activity increase between the 1.7- and 2.4-fold under screening conditions could be confirmed by HPLC. Sequencing identified the amino acid replacements H171L, Q166R/L271M and S168R/M286I/Q315E, respectively, for the selected clones. For the identification of the residues which cause the increased activity in case of the double and triple mutant all observed exchanges were introduced separately into CYP11B1 Pa1 by site-directed mutagenesis and analyzed for their whole-cell activity in microtiter plates using HPLC. Their activities regarding cortisol formation in comparison with the CYP11B1 Pa1 enzyme are shown in Figure 8.

The amino acid exchange Q166R, which occurred in the double mutant Q166R/L271M, reduced the CYP11B1 activity to about 50% when introduced individually. The removal of this unfavorable exchange leading to the single mutant L271M increased activity of CYP11B1 Pa1 approximately 3.4-fold. Two of the exchanges of the triple mutant, S168R and Q315E, did not show any or only a slightly beneficial effect on product formation, while the third exchange, M286I, alone enhanced product formation by a factor of 2.7 compared with CYP11B1 Pa1, which represents an additional slight increase compared with the parental triple mutant. The best mutant, L271M, was chosen for subsequent experiments. No significant differences in expression level in the microtiter plate were observed between CYP11B1 Pa1 and L271M, which were synthesized with 57 and 69 nmol∗L^−1^, respectively. This excludes enzyme stability as underlying cause for the enhanced product yield.

### Large-scale cortisol production by the selected CYP11B1 mutant

**Figure 9:**
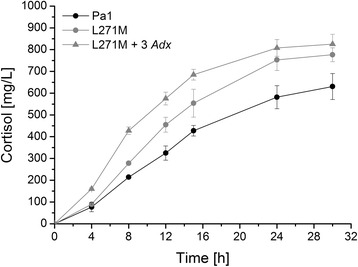
**Time-dependent whole-cell cortisol formation by the parental CYP11B1 enzyme (Pa1) and the selected mutant L271M in shaking flasks.** Pa1 (black circles) was expressed from the Twin_11B1 plasmid carrying 1 *Adx* copy, while L271M was expressed from plasmids with either 1 *Adx* (grey cirlces) or 3 *Adx* (grey triangles) copies. Reactions were performed after a 21-h expression period in TB medium with resting cells in presence of 6% DMSO and 3 mM 11-deoxycortisol. Extracted steroids were analyzed by RP-HPLC. Values represent the mean of three conversion experiments conducted in parallel with respective standard deviation.

In order to verify the reliability of the microtiter plate screening for activities in larger scale, mutant L271M was analyzed for its time-dependent capacity of cortisol formation with resting cells in shaking flasks as described in Material and methods. As expected, the mutant turned out to be more productive than its parent CYP11B1 Pa1 and its application enabled an increase of the final product concentration from 631 to 777 mg∗L^−1^ (Figure 9). The expression level of L271M in shaking flasks was ascertained as approximately 240 nmol∗L^−1^ and is thus comparable to the expression level of CYP11B1 Pa1. Moreover, we combined the mutant in a plasmid with 3 *Adx* copies, as this number maximized Adx synthesis in the preceding experiments. This combination revealed an additive effect during the initial phase of the reaction and the productivity of L271M over the first 12 h was stimulated by the enhanced Adx availability in a dimension comparable to the effect of 3 *Adx* copies on CYP11B1 Pa1, leading from 38 to 48 mg∗L^−1^∗h^−1^ (Figure 9).

## Discussion

**Figure 10:**
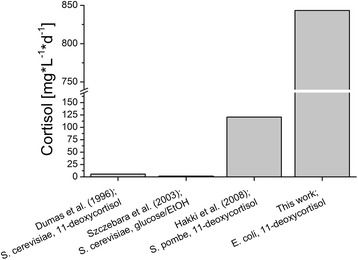
**Comparison of the volumetric productivities of publically available CYP11B1 dependent whole-cell biocatalysts for cortisol production.** The respective publications as well as the host organisms and the substrates are indicated.

CYP11B1, which synthesizes cortisol from 11-deoxycortisol in the human adrenal cortex, also exposes a great potential as biocatalyst in the industrial synthesis of cortisol, a pharmaceutically and thus commercially important steroid due to its anti-inflammatory and immunosuppressive effects. In this work, the successful reconstitution of a CYP11B1 system in a recombinant *E. coli* whole-cell biocatalyst, which is capable of forming cortisol from 11-deoxycortisol by 11β-hydroxylation, is presented. Because of the selectivity of 11-deoxycortisol hydroxylation by CYP11B1 in combination with a high volumetric productivity and the absence of side-product formation by *E. coli*, the developed system provides distinct advantages over other bioprocesses that have been established for cortisol production. Current industrial synthesis from 11-deoxycortisol by means of an 11β-hydroxylation via biotransformation by the fungus *C. lunata* [4] is accompanied by side-product formation [6], while alternative systems in recombinant yeast suffer from poor efficiencies [17-20]. Figure 10 compares the volumetric productivities [mg∗L^−1^∗d^−1^] of publically accessible systems for cortisol production by CYP11B1 dependent biotransformation. Our new *E. coli* based system, which enabled a maximum volumetric productivity of 843 mg∗L^−1^∗d^−1^, exhibits a productivity which is nearly one order of magnitude higher than the best value published. In order to realize this, strategies have been developed to successfully target factors which frequently limit the application of P450s in industrial biocatalysis.

Recent studies hint at a favorable effect of N-terminal replacements of hydrophobic amino acids by positively charged ones for the expression and stability of eukaryotic P450s in *E. coli* [30,38]. The introduction of a hydrophilic amino acid near the N-terminus of CYP11B1 by site-directed mutagenesis of position 23 from glycine to arginine greatly enhanced the expression level, while maintaining catalytic activity, and thus improved initial productivity as well as the final product yield by a factor of 2.6.

After successfully increasing CYP11B1 expression, our second aim was an optimization of the electron flux from cellular NADPH towards CYP11B1 by engineering the redox chain which is reconstituted in *E. coli* by co-expressing AdR and Adx with the P450 from a tricistronic plasmid. Especially the electron transfer from Adx to the P450 is known to be an activity limiting step in P450 catalysis, which can be at least partially rescued by truncation and mutagenesis of Adx [31,32] or by increasing the Adx availability in the system which could already be demonstrated for CYP11B1 in in-vitro experiments with purified enzymes [21,39], as well as in recombinant cell cultures [40] and yeast [19]. In-vitro studies proof a dependency of CYP11B1 activity on Adx concentration following the Michealis-Menten equation at a stable AdR concentration [21] and describe that upon excess of Adx the maximum CYP11B1 hydroxylation activity is already achieved at molar ratios of AdR/CYP11B1 lower than 1 [41-43]. Additionally, investigations of a *class I* P450 system reconstituted in *E. coli* show a ratio for P450:ferredoxin:reductase of 1:6:1 under expression conditions optimized for substrate hydroxylation with CYP105A1 [44]. Therefore, we decided to engineer the ratio of *CYP11B1* to *Adx* expression and introduced additional copies of the Adx cDNA into the polycistronic expression unit in order to enhance *Adx* expression on transcriptional level and to reduce the rate-limiting factor of Adx availability in the *E. coli* biocatalyst. The approach succeeded to improve the productivity to a maximum possible within the polycistronic transcriptional strategy. Initial productivity was greatly accelerated and the final product concentration was increased by a factor of 1.4 at the maximum Adx level. This indicates that the limitations of the whole-cell activity caused by a reduced Adx availability were successfully overcome by the presented approach. Moreover, the high Adx concentration might have a general positive effect on the viability of the whole-cells as the [2Fe-2S]-cluster can function as a scavenger by trapping reactive oxygen species [45] which can be formed in the course of the P450 catalytic cycle [11].

In a parallel approach for the optimization of the whole-cell activity, new CYP11B1 variants with an increased activity of cortisol formation from 11-deoxycortisol were generated. Directed evolution, which consists of one or several cycles of enzyme mutagenesis, screening for the desired enzyme properties and selection of favorable mutants, represents a classical tool for such kind of enzyme engineering towards improved catalytic efficiencies, reduced uncoupling, altered selectivity or substrate specificity [46]. However, the crucial step in the establishment of a system for directed evolution is the development of a screening assay which enables a sensitive and accurate selection of the desired enzyme features with a high throughput. In order to meet these criteria, we performed a down-scale of the steroid-converting *E. coli* system to microtiter plates and employed a fluorescence based activity assay [35] in a robot-assisted manner. This assay has already been successfully applied for the improvement of the catalytic activity of CYP106A2 from *Bacillus megaterium* towards its steroidal substrates 11-deoxycortisol and progesterone [37,47]. It is premised on the fluorescence developed by steroids in an acidic, hygroscopic environment whose intensity can vary between substrate and product of a hydroxylation reaction. This is true for the transformation of 11-deoxycortisol to cortisol in the presented *E. coli* system with increasing fluorescence intensity upon formation of cortisol which proofs the applicability of the assay for the detection of activity enhanced mutants. Because of the little information about structure-activity relation of adrenocortical P450s in the literature, we conducted a random PCR mutagenesis of the entire CYP11B1 gene and examined the arising mutant library with the fluorescence screening test. We were able to select 3 mutants, H171L, Q166R/L271M and S168R/M286I/Q315E, that exhibited an approximately 2-fold increased activity in the microtiter scale and retained selectivity as shown by HPLC measurements. The activity improvements measured via fluorescence screening in the microtiter plates were supported by HPLC analysis underlining reliability of the screening procedure. The predicted localizations of mutated residues are summarized in Table 1.

**Table 1:** **Localization of amino acid exchanges of CYP11B1 mutants with increased activity**

**Amino acid exchange**	**Localization**
H171L	E’-helix, protein surface
Q166R	E’-helix, protein surface
L271M	H-helix, protein surface
S168R	E’-helix, protein surface
M286I	I-helix
Q315E	J-helix, protein surface

Localization of the respective residues discovered during the screening of a random CYP11B1 library for mutants with increased 11β-hydroxylation activity towards 11-deoxycortisol was deduced from the latest homology model of CYP11B1 [25].

**Figure 11:**
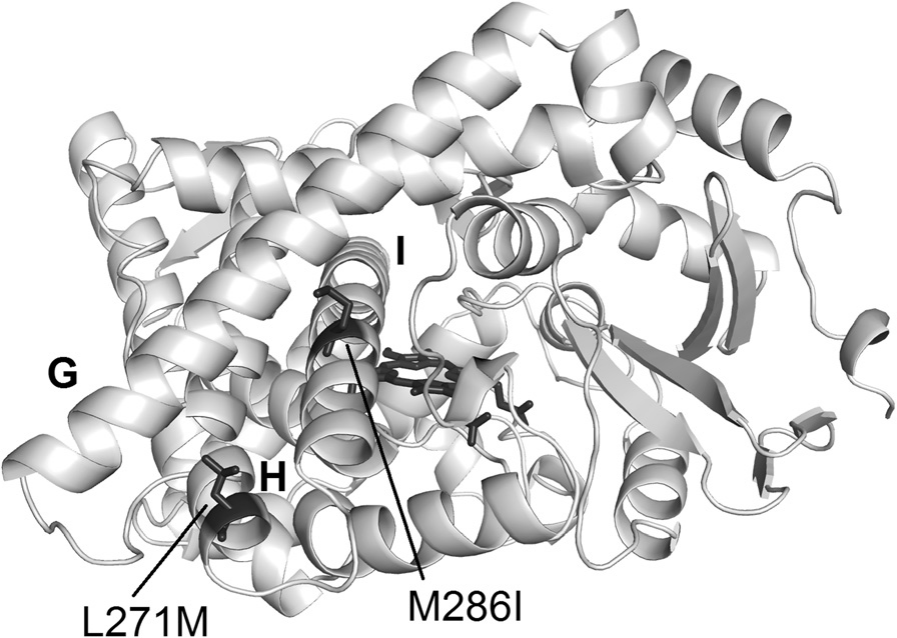
**Localization of amino acid exchanges of CYP11B1 that increase hydroxylation activity towards 11-deoxycortisol.** L271M is located on the protein surface in the H-helix, while M286I resides in the I-helix. Helices are labeled with their eponymous letter. The structure represents the latest homology model [25] and figures were created with PyMOL Builds. Mutated amino acids and the heme are highlighted in black.

The subsequent individual analysis of amino acid exchanges from the double and triple mutant revealed activity impairing effects of mutations that introduce a positive charge into the E’-helix (S168R, Q166R), while the elimination of a potentially positively charged residue (H171L) increases the CYP11B1 activity. The removal of the unfavorable mutations further enhanced cortisol formation up to 3.4-fold compared with Pa1, when using L271M or M286I. L271M, a conservative exchange which is predicted to be localized in the H-helix on the protein surface (Figure 11), was identified as the exchange that contributes most efficiently to CYP11B1 activity. As the H-helix represents the link between the I-helix which traverses the active site and the mobile F/G-loop, which is involved in substrate access to the active site [48], the slight alteration in the residue’s physicochemical properties might positively influence the flexibility of CYP11B1. M286I, the second activity increasing amino acid replacement, resides in the I-helix (Figure 11). It is not part of the active site pocket but can be assumed to strengthen structural integrity of this core element. Q315E leads to the introduction of a charged group in the J-helix on the protein surface and does not have significant impact on the CYP11B1 activity which identifies M286I as determining mutation for the selection of the triple mutant. An upscale of the system from microtiter plates to shaking flasks using the most efficient mutant, L271M, could successfully reproduce the improvement of the whole-cell activity and enhanced the initial productivity as well as the final product concentration in comparison to CYP11B1 Pa1. The proposed screening procedure can thus be regarded as reliable for the optimization of large-scale processes by laboratory evolution. The mutagenesis approach can be combined with the strategy of engineering the redox partner co-expression leading to additive effects (Figure 9). However, the activity of all systems flattens after approximately 24 h and cannot be rescued with the approaches presented in this work. This points at the necessity of further optimization on the levels of process and strain engineering.

## Conclusions

Taken together, we report the establishment of an *E. coli* based biocatalyst for cortisol production by a heterologous CYP11B1 system, which enables a maximum productivity of 0.84 g∗L^−1^∗d^−1^ under simple shaking flask conditions and thus clearly meets efficiency requirements for potential application in the pharmaceutical industry [49,50]. In total, our optimization approaches could increase the cortisol yield by a factor of 3.7. The presented strategy to overcome activity limits due to low protein ratios of Adx to CYP11B1 can be transferred to other biotechnologically interesting P450 redox chains in whole-cell application as Adx represents an efficient electron transfer partner not only for mitochondrial but also for microsomal and bacterial P450s [31,51]. The successful establishment of an accurate and fast screening system for CYP11B1 activity in combination with new structure-activity insights from the first mutant generation can be used for further directed evolution of the enzyme [52]. The system might additionally be applicable for the screening of CYP11B1 inhibitors, which can be important drugs for the treatment of for example Cushing’s syndrome [53].

## Material and methods

### Chemicals and enzymes

All chemicals and reagents were purchased from standard sources in the highest purity available. Restriction enzymes were obtained from New England Biolabs (Ispwich, MA, USA), *Pfu* polymerase from Promega (Madison, WI, USA), and FastLink Ligase from Epicentre Biotechnologies (Chicago, IL, USA).

#### Bacterial strains and cultivation

Plasmid construction was performed with *E. coli* TOP10F’ (*F-mcrA (mrrhsdRMS-mcrBC) f80lacZDM15 DlacX74 deoR recA1 araD139 (ara-leu)7697 galU galK rpsL (StrR) endA1 nupG*). All experiments involving protein expression and steroid conversion were conducted with *E. coli* C43(DE3) (*F– ompT gal hsdSB (rB- mB-) dcm Ion* λ). Transformation was carried out by electroporation and transformed cells were stored on agar plates supplemented with the appropriate antibiotics (100 μg/mL ampicillin and/or 50 μg/mL kanamycin) at 4°C.

### Plasmid construction and mutagenesis

All methods of molecular biology were performed according to standard protocols described by [54]. The plasmid Twin_11B1 served as template for the preparation of all further plasmids. It is based on the pET-17b expression vector (MerckMillipore Novagen, Darmstadt, Germany), which enables a selection on ampicillin containing medium, and carries the cDNAs of human CYP11B1 cloned into the vector via NdeI/HindIII, bovine AdR via HindIII/KpnI and bovine Adx_1–108_ via KpnI/EcoRI in a polycistrionic transcription unit [55]. CYP11B1 was modified for expression in *E. coli* as described by [21]. Additionally, residue 29 in the *E. coli* adapted sequence (corresponding to residue 52 in the full length sequence) is mutated from leucine to methionine taking previous activity studies in recombinant fission yeast [19] into account. All utilized primers are shown in Additional file 1: Table S1.

### Insertion of additional gene copies

In order to consecutively insert additional copies of the Adx cDNA into the Twin_11B1 plasmid behind the pre-existing copy, the following strategy, which takes advantage of restriction site compatibility of EcoRI and MfeI, was pursued referring to [34]. In a first step, Twin_11B1 was used as a template to amplify the Adx cDNA by PCR including its ribosomal binding site and to introduce MfeI and XhoI restriction sites at the 5′ and 3′ end, respectively. The PCR product was then digested by MfeI and XhoI and ligated into the EcoRI/XhoI digested Twin_11B1 plasmid resulting in a plasmid carrying 2 Adx copies. In additional cycles of restriction and ligation further copies were inserted. In subsequent cloning attempts inserts of different Adx copy numbers could then be introduced into new plasmids by restriction and ligation via KpnI and EcoRI.

### Site directed mutagenesis of CYP11B1

Targeted exchange of single amino acids was undertaken by QuikChange® mutagenesis with *Pfu* polymerase following manual instructions form Agilent Technologies (Santa Clara, USA).

### Random mutagenesis of CYP11B1

Random mutagenesis of CYP11B1 was conducted by error prone PCR employing the GeneMorph II random mutagenesis kit (Stratagene, La Jolla, CA, USA). pET17b_hCYP11B1, which contains the modified cDNA of human CYP11B1 between the NdeI and HindII restriction sites of its multiple cloning site, was used as a template for the amplification of CYP11B1 with the standard primers T7 and T7term. Parameters for an average mutation frequency of 0–3 mutations per kb were chosen according to the manufacturer’s protocol. The PCR product was digested by NdeI and HindIII and ligated into the likewise digested Twin_11B1 plasmid.

### Whole-cell biocatalysis in shaking flasks

#### Protein expression

The synthesis of CYP11B1, AdR and Adx in *E. coli* took place as co-expression with the chaperone genes *GroEL* and *GroES* to ensure proper folding. Protein synthesis was carried out in 2 L Erlenmeyer flasks containing 150 mL TB medium (24 yeast extract technical, 12 g peptone, 4 mL glycerol, 4,62 g KH_2_PO_4_, 25 g K_2_HPO_4_ and distilled water ad. 1 L) supplemented with 100 μg/mL ampicillin and 50 μg/mL kanamycin. The main culture was inoculated from an overnight culture of *E. coli* C43(DE3), that had been freshly co-transformed with the respective variant of Twin_11B1 and the chaperone vector pGro12 (kanamycin resistance and arabinose inducible promoter) [23], and was grown at 37°C and 210 rpm (Excella 25 shaker incubator, New Brunswick Scientific, Eppendorf, Ensfield, CT, USA). When an OD_600 nm_ of 0.5 was reached expression was induced by addition of 1 mM IPTG, 4 mg/mL arabinose, 1 mM of the heme precursor δ-aminolevulinic acid and 50 μg/mL ampicillin. Cultures were further incubated at 27.5°C and 200 rpm for 21 h.

### Steroid conversion with resting cells

Subsequent to the expression period cultures were harvested by centrifugation (3200 *g*, 10 min, 18°C) and cells were washed in 50 mM potassium phosphate buffer (pH 7.4). Steroid conversion took place at 27.5°C and 170 rpm in 300-mL baffled flasks using 25 mL of a cell suspension of 25 g wet cell weight (wcw) per L in 50 mM potassium phosphate buffer (pH 7.4) supplemented with 1 mM IPTG, 4 mg/mL arabinose, 1 mM δ-aminolevulinic acid, 50 μg/mL ampicillin and 2% glycerol. The substrate 11-deoxycortisol (17,21-dihydroxypregn-4-ene-3,20-dione) was added from a stock solution in either EtOH, DMSO, 22.5% (m/vol) 2-hydroxypropyl-β-cyclodextrin or a 1:1 (vol:vol) mixture of EtOH and polyethyleneglycol-400. Each agent was added to the culture in a final concentration of 6% (vol/vol). Samples were taken at defined time points.

### Reversed phase HPLC analysis

For product quantification via HPLC, samples were extracted twice with one volume of chloroform. After evaporation of the organic solvent remaining steroids were suspended in acetonitrile and separated on a Jasco reversed phase HPLC system of the LC900 series (Jasco, Groß-Umstadt, Germany) using a 4.6 m × 125 mm NucleoDur C18 Isis Reversed Phase column (Macherey-Nagel, Düren, Germany) with an acetonitril/water gradient (Phase A: 10% acetonitrile, Phase B: 100% acetonitril; 0 min 20% B, 5 min 20% B, 13 min 40% B, 20 min 80% B, 21 min 80% B, 22 min 20% B, 30 min 20% B) at 40°C and a flow rate of 0.8 mL/min. Steroid pattern was monitored by an UV/Vis detector (UV-2 075 Plus, Jasco) at 240 nm.

### Screening for improved CYP11B1 activity in microtiter plates

Protein expression and steroid conversion in microtiter plates as well as a fluorescence assay for the selection of CYP11B1 mutants with an improved hydroxylation activity towards 11-deoxycortisol was performed as previously reported [36], but TB medium was additionally supplemented with 50 μg/mL kanamycin and 4 mg/mL arabinose to ensure chaperone synthesis from pGro12 and did not contain a salt solution.

### Analysis of protein expression

#### Cell lysis

For the analysis of CYP11B1 expression levels cells were harvested by centrifugation (4500 *g*, 20 min, 4°C), suspended in lysis buffer (50 mM potassium phosphate buffer (pH 7.4), 500 mM sodium-acetate, 0.1 mM EDTA, 1.5% sodium-cholate, 20% glycerol, 1.5% Tween 20, 0.1 mM phenylmethylsulfonylfluorid and 0.1 mM dithioerythritol) and disrupted with an ultrasonic homogenizer (Sonopuls HD 3200, Bandelin, Berlin, Germany). Cell debris were removed by ultracentrifugation (30000 g, 30 min, 4°C; hinac CP75, Hitachi, Tokyo, Japan) and the supernatant was surveyed for recombinant proteins.

### Determination of cytochrome P450 concentration

P450 concentration was determined by CO-difference spectroscopy using a molar extinction coefficient of 91 mM^−1^ cm^−1^ as described by [56].

### Western blot analysis of Adx synthesis

Cell pellets from 90 μL of the 25g_wcw_/L cell suspension were suspended in 100 μL of SDS loading buffer (1 M Tris–HCl (pH 6.8), 40% glycerol, 20% SDS, 8% β-mercaptoethanol, 0.1% bromphenol blue) and boiled for 10 min in a water bath. Aliquots of 12 μL were separated by SDS-PAGE according to [57]. 8 μL of a 5 μM solution of purified bovine Adx_4–108_ in SDS loading buffer were applied as positive control and Protein Marker IV from PEQLAB (Erlangen, Germany) served as molecular weight standard. Proteins were blotted onto a hybond™ ECL™ nitrocellulose membrane (Amersham, GE Healthcare, UK) with the help of a semi-dry transfer system (Trans-Blot SD, Bio-Rad, Munich, Germany) and the membrane was blocked by incubation with 3% milk powder in TBS (50 mM Tris–Cl pH 7.4, 200 mM NaCl, 0.1% Tween 20) overnight. The membrane was washed 3 times for 10 minutes in fresh TBS and was incubated for 2 h with the respective polyclonal antiserum from rabbit diluted in TBS. Subsequent to 3 further washing steps in TBS the membrane was incubated for 2 h with a dilution of the horseradish peroxidase-linked goat anti rabbit IgG secondary antibody (Dako, Glostrup, Denmark) in TBS. After washing the membrane 3 times for 5 minutes with PBS (10 mM potassium phosphate buffer pH 7.4, 150 mM NaCl) staining of the antigen-antibody-complexes took place by adding 5 mg 4-chloro-1-naphtol dissolved in 2 mL ethanol and 10 μL 30% H_2_O_2_ in 25 mL PBS. Relative intensity of the protein bands was measured with Image Lab 3.0 from BioRad (München, Germany).

## Additional file

Additional file 1: Table S1. Primers used in this work with sequence and purpose of application. Restriction sites are marked with bold letters, introduced nucleotide exchanges are underlined.

## Abbreviations

AdR: Adrenodoxin reductase
Adx: Adrenodoxin
CYP11B1: 11β-hydroxylase
*E. coli*: *Escherichia coli*
