# Supplementary material for: A recombinant CYP11B1 dependent Escherichia coli biocatalyst for selective cortisol production and optimization towards a preparative scale
Source: Microb Cell Fact. 2015 Feb 25;14:25. doi: 10.1186/s12934-015-0209-5 (PMC4347555; doi:10.1186/s12934-015-0209-5)
Supplement: Additional file 1: Table S1. — Primers used in this work with sequence and purpose of application. Restriction sites are marked with bold letters, introduced nucleotide exchanges are underlined. [file 12934_2015_209_MOESM1_ESM.doc]

**Additional file 1: Table S1: Primers used in this work with sequence and purpose of application. Restriction sites are marked with bold letters, introduced nucleotide exchanges are underlined.**

| **Name** | **Sequence 5’-3’** | **Application** |
| --- | --- | --- |
| **F_11B1_R23** | GTCGTCCACGCAACCGTTGGCTGCGTATGCTGCAG | Forward primer for introduction of G23R in 11B1 |
| **R_11B1_R23** | ACGGTTGCGTGGACGACGTGGCATGGCTTCAAATGG | Forward primer for introduction of G23R in 11B1 |
| **F_MfeI_Adx** | TCG**CAATTG**AAGAAGGAGATATACCATGAGCAGCTC | Forward primer for the amplification of Adx including RBS and 5’MfeI site |
| **R_XhoI_Adx** | TCA**CTCGAG**GCAGAATTCTTAAGGTACTCGAACAG | Reverse primer for the amplification of Adx including a 3’XhoI site |
| **F_11B1_Q166R** | CCTGGACGTCCGGCCCAGCATCTTCCACTACACC | Forward primer for introduction of Q166R in 11B1 |
| **R_11B1_Q166R** | AAGATGCTGGGCCGGACGTCCAGGGTCAGG | Reverse primer for introduction of Q166R in 11B1 |
| **F_11B1_S168R** | CCAGCCCAGAATCTTCCACTACACCATAGAAGCC | Forward primer for introduction of S168R in 11B1 |
| **R_11B1_S168R** | GAAGATTCTGGGCTGGACGTCCAGGGTCAG | Reverse primer for introduction of S168R in 11B1 |
| **F_11B1_H171L** | CATCTTCCTCTACACCATAGAAGCCAGCAACTTGGC | Forward primer for introduction of H171L in 11B1 |
| **R_11B1_H171L** | TCTATGGTGTAGAGGAAGATGCTGGGCTGGACGTC | Reverse primer for introduction of H171L in 11B1 |
| **F_11B1_L271M** | GGAGCTTATGTTGAACGCGGAACTGTCGCCAGATGCC | Forward primer for introduction of L271M in 11B1 |
| **R_11B1_L271M** | CGCGTTCAACATAAGCTCCGCCACGATGCTGGTGTAC | Reverse primer for introduction of L271M in 11B1 |
| **F_11B1_M286I** | ACTCTATAGAACTCACAGCAGGGAGCGTGGACACGAC | Forward primer for introduction of M286I in 11B1 |
| **R_11B1_M286I** | TCCCTGCTGTGAGTTCTATAGAGTTGGCCTTGATGG | Reverse primer for introduction of M286I in 11B1 |
| **F_11B1_Q315E** | ACGTGCAGGAGGCCCTGCGCCAGGAG | Forward primer for introduction of Q315E in 11B1 |
| **R_11B1_Q315E** | AGGGCCTCCTGCACGTTGGGGTTCCG | Reverse primer for introduction of Q315E in 11B1 |
